# The Role of Need for Cognition and Its Interaction with Fluid Intelligence in the Prediction of School Grades in Primary School Children

**DOI:** 10.3390/jintelligence13080094

**Published:** 2025-07-28

**Authors:** Anke Hufer-Thamm, Sebastian Bergold, Ricarda Steinmayr

**Affiliations:** 1 Department of Psychology, TU Dortmund University, Emil-Figge-Str. 50, 44227 Dortmund, Germany; 2 Department of Educational Psychology, University of Rostock, August-Bebel-Str. 28, 18051 Rostock, Germany; sebastian.bergold@uni-rostock.de

**Keywords:** need for cognition, fluid intelligence, academic achievement, primary school

## Abstract

Fluid intelligence and need for cognition are relevant predictors of school grades and might also interact in the prediction of grades. We examined the independent predictive values of fluid intelligence and need for cognition as well as their interaction for math and German grades and changes therein in a sample of 565 German primary school children (298 girls, 261 boys, 6 with no gender specified; *M*_age_ = 8.40, *SD* = 0.59). Parental education was considered a control variable. Cross-sectional analyses showed that both intelligence and need for cognition were uniquely related to grades. However, in the latent change score analyses, fluid intelligence, but not need for cognition, was related to change in math grades, but not in German grades, and only when parental education was not considered as a control variable. We found no interaction effects between fluid intelligence and need for cognition. The findings imply that the need for cognition might not play a comparably relevant role for school grades in primary school as it has been shown in previous studies focusing on secondary or tertiary education.

## 1. Introduction

Intelligence is evidently one of the strongest single predictors of academic achievement (e.g., Mammadov 2022; Steinmayr et al. 2014). It explains about 25% of the variance in different academic achievement indicators (e.g., Roth et al. 2015) compared to about 16% (top) of other important predictors like ability self-concept (Möller et al. 2009). Cognitive abilities set the grounds for what an individual is capable of achieving (Kuncel et al. 2004), also with regard to school grades, which is one of the most relevant indicators of academic achievement (Steinmayr et al. 2014). Next to external factors such as socioeconomic status of the family (Steinmayr and Kessels 2024; Weidinger et al. 2020), a variety of personality and motivational variables also play a role in shaping the development of school grades. Among those, the investment trait Need for Cognition (NFC), defined as the stable tendency to seek out and enjoy tasks that are cognitively challenging (Cacioppo et al. 1996), has been shown to have substantial relations to school grades and other achievement indicators (e.g., Preckel 2014; Strobel et al. 2024). This previous evidence, however, mostly stems from studies focusing on pupils in secondary or tertiary education. NFC has been investigated much less frequently in primary school children, especially regarding its connection to educational achievement and its incremental predictive value beyond intelligence. However, primary school age is a crucial phase for both intellectual and motivational development (Bergold and Steinmayr 2024). Primary school children usually start their school career with a strong motivation to learn, accompanied by overly optimistic ability self-perceptions, both of which decrease during primary school (e.g., Jacobs et al. 2002). This decline is due to increased social comparisons and other developmental changes (Weidinger et al. 2019). At the same time, fluid as well as crystallized cognitive abilities develop, while their relative stabilities increase, setting the ground for the pupils’ later abilities (e.g., Bloom et al. 2008; McCall 1977). Thus, both learning motivation and cognitive abilities undergo rapid change in the primary school years. Primary school age could thus be a particularly suitable time for fostering investment traits such as NFC, if investment traits are shown to have an effect on the development of educational success. To investigate this research question, longitudinal studies on pupils’ academic development are needed. However, only a few studies in the field have used information on educational achievement from more than one measurement occasion.

Furthermore, intelligence and NFC might not only exert independent influence on school grades, but might also interact in the prediction of school grades and the changes therein. Theoretically, it might be that children benefit from being intelligent *and* scoring high on NFC (synergistic interaction) or that less intelligent pupils might compensate for their lower cognitive abilities with high NFC in achieving good school grades (compensatory interaction). In the present study, we were interested in the main effects of fluid intelligence (Gf) and NFC, the incremental effects of NFC, and potential interactions of Gf and NFC in predicting (changes in) school grades in the subjects math and German (first language). A sample of primary school children was assessed in third and fourth grade and analysed by means of hierarchical regressions and latent change score models.

### 1.1. Intelligence and Academic Achievement

Gf is defined by classic and modern theories as cognitive processing capabilities used to solve novel problems to which acquired knowledge cannot be applied (Cattell 1963; Horn and Cattell 1966). By contrast, crystallized intelligence (Gc) refers to accumulated cultural knowledge. Intelligence has been shown to be the most relevant psychological predictor of academic achievement (Mammadov 2022), as it provides the basis for what an individual can achieve academically.

Academic achievement can be measured via various indicators, which all display different aspects of academic achievement, for instance, the highest school leaving certificates, standardized tests, or school grades. The latter, often reflected by the grade point average (GPA), are among the most widely used performance outcomes in educational research, due to their crucial relevance for the pupils’ future academic and occupational paths (e.g., Steinmayr et al. 2014). The meta-analysis by Roth et al. (2015) showed a population correlation between intelligence and school grades of ρ = 0.54. According to their results, the correlation is highest for mixed intelligence tests and is lowest in primary school compared to higher educational levels. However, in Germany, it tends to be the other way round, as after fourth grade, most pupils are tracked in different school forms depending on their academic achievement, which decreases variance within school tracks, leading to lower correlations (see Steinmayr and Kessels 2024).

### 1.2. Need for Cognition and Academic Achievement

NFC has been defined by Cacioppo et al. (1996) as a “stable individual [difference] in people’s tendency to engage in and enjoy effortful cognitive activity” (p. 197). As such, NFC has been conceptualized as a personality trait, but it also has conceptual proximity to motivational constructs such as intrinsic motivation (towards cognitively challenging tasks). While intelligence describes what an individual *can* do with regard to cognitively challenging situations, NFC refers to what an individual *usually* does in these kinds of situations (Strobel et al. 2019). NFC is assigned to a group of personality characteristics called investment traits, which describe how individuals seek out (or avoid) intellectual challenges and how much cognitive effort they spend on these challenges once they encounter them (von Stumm and Ackerman 2013). Within the intellect model proposed by Mussel (2013), NFC is primarily allocated in the process dimension *seek* and in the operation dimension *think*, as it describes how people seek out tasks that require effort in thinking. According to the hierarchical model by von Stumm and Ackerman (2013), NFC is understood as a core investment trait (next to Typical Intellectual Engagement), which underlines its theoretical relevance.

The inherent features of NFC should theoretically be advantageous for academic achievement, irrespective of intelligence. Individuals scoring high on NFC have often been referred to as ‘chronic cognizers’ (Cacioppo et al. 1996), who tend to process information more deeply. By definition, they enjoy cognitive challenges and thus prefer complex over easy tasks and invest more effort into problem-solving and knowledge acquisition (von Stumm and Ackerman 2013). Within the elaboration likelihood model, NFC is connected to the usage of the central rather than the peripheral route in information processing (Petty and Cacioppo 1984).

Prior empirical evidence has indeed aligned with theoretical considerations. A recent meta-analysis (Liu and Nesbit 2024) demonstrated moderate associations between NFC and academic achievement (*r* = 0.20). This relationship was stronger in higher grade levels. In a sample of German pupils at the beginning of secondary school, NFC predicted math grades beyond intelligence (Preckel 2014). Lavrijsen et al. (2023) came to similar results, showing that NFC was related to GPA in a sample of Flemish 7th-graders. However, changes in school grades were not considered in either of these studies. Thus far, to our knowledge, two studies investigated the role of NFC in explaining variance in academic achievement in primary school children. In a Finnish sample, NFC had very small relations to school grades in grade 3 (Luong et al. 2017). Measures of cognitive potential were, however, not included in the regression analyses, so that the increment of NFC was not examined. Keller et al. (2019) found that NFC explained variance in math and language grades in a Finnish and a Luxemburgish sample of primary school children beyond other motivational variables. In a German sample of third- and fourth-graders within the same study, NFC only had incremental predictive value for German grades, not for math grades.

The evidence from longitudinal studies is mixed: In a sample of German secondary school pupils, GPA predicted change in NFC in cross-lagged panel models from grade 11 to 12 (Steinmayr et al. 2023) beyond the effect of intelligence, which had been included as a control variable. NFC, on the other hand, did not predict GPA. Strobel et al. (2024), however, demonstrated that latent change of GPA was predicted by NFC (β = 0.24) above motivational variables such as ability self-concept in a study also relying on German secondary school pupils, while intelligence was not included.

### 1.3. Possible Interactions Between Need for Cognition and Intelligence

Besides the main effects of intelligence and NFC, these traits could also interact in the explanation of variance in school grades and in the prediction of changes therein. From the perspective of investment theories (Ackerman 1996; Ziegler et al. 2012), investment traits such as NFC influence the extent to which cognitive abilities are invested in academic achievement. Theoretically, two types of interaction are possible: The first one is the *synergistic* form, which implies that intelligence and NFC would reinforce each other. That is, the more intelligent pupils would benefit more from scoring high on NFC, because being eager for intellectual challenges helps them to fully use their cognitive potential and to achieve good grades. The second interaction form—the *compensatory* interaction—refers to intelligence and NFC compensating each other. In this scenario, the less smart children can make up for this lack by seeking out and engaging in intellectually stimulating tasks.

Only a few studies have explored interactions between intelligence and NFC to date, and their results are mixed. Strobel et al. (2019) found a compensatory interaction (β = −0.12, ΔR^2^ = 3.0%) of Gf and NFC in the retrospective prediction of GPA in the university entrance diploma (Abitur) of German undergraduate psychology students. On the other hand, NFC and intelligence did not interact in the (cross-sectional) prediction of GPA in Flemish 7th-graders (Lavrijsen et al. 2023) nor in predicting change in Gc in German 7th-graders (Scherrer et al. 2024). Research on constructs related to NFC has furthermore yielded evidence that NFC and interest (but not openness) interacted in a synergistic way when the outcomes were reading and math competencies (Lechner et al. 2019). Openness interacted with figural reasoning in the (cross-sectional) prediction of school grades in Chinese secondary school pupils (Zhang and Ziegler 2015). However, it did not interact with intelligence in the (cross-sectional) prediction of GPA in German high school pupils (Bergold and Steinmayr 2018). Only when the facet *Actions* was analysed, the authors found an interaction with intelligence. Besides the scarcity of longitudinal analyses, no study has ever considered an interaction between intelligence and NFC in primary school samples.

### 1.4. Present Study

In sum, to date, we know of two cross-sectional studies on NFC and its relation to academic achievement in primary school, which is a crucial phase for intellectual development, despite the relation between NFC and achievement tending to be lower in younger age (Liu and Nesbit 2024). Thus, little is known about the incremental effect of NFC on achievement beyond intelligence at this age. The scarcity of studies focusing on NFC and academic achievement that comprise more than one measurement occasion and that are thus capable of analysing changes in achievement is another gap in the existing research. Possible interaction effects between NFC and intelligence have rarely been investigated, and no study has done so for primary school.

In our study, we aimed to address these research gaps and investigated the associations between NFC, fluid intelligence, and grades in math and German in a sample of German third-graders who were followed over two measurement occasions covering one year. This enabled us not only to examine whether NFC was related to school grades but also to analyse whether it predicted potential changes in these school grades beyond fluid intelligence. Furthermore, we focused on possible interaction effects of NFC and intelligence in the prediction of (changes in) school grades.

Based on theoretical reflections and previous empirical results, we tested two hypotheses (H). With regard to potential interaction effects, a synergistic and a compensatory interaction would be reflected in opposite signs. Since both interaction forms would be plausible, we additionally investigated one exploratory research question (RQ).

**H1a.**  *Gf is positively related to German and math grades.*

**H1b.**  *Gf positively predicts change in German and math grades.*

**H2a.**  *NFC is positively related to German and math grades beyond Gf.*

**H2b.**  *NFC positively predicts change in German and math grades beyond Gf.*

**RQ1:**  *Do Gf and NFC interact in the prediction of (change in) German and math grades?*

Since parental socio-economic status influences teachers’ judgments (including grades; e.g., Weidinger et al. 2020), but also their children’s intelligence (Rindermann and Ceci 2018) and NFC (Colling et al. 2022), we considered parental education (i.e., a proxy of the family’s socio-economic status) as a control variable. Thus, controlling for parental education is essential to eliminate the possibility that it accounts for the relationships among the other three variables.

## 2. Materials and Methods

### 2.1. Procedure and Participants

Data collection was part of a project following primary school children over two measurement times (Bergold and Steinmayr 2024). The study was conducted in accordance with the Declaration of Helsinki and approved by the ethics committee of the investigators’ university. Written informed consent was obtained from the children’s parents. Data for the first measurement point (t_1_) was gathered between August and October 2021, right after the start of third grade. We considered the beginning of third grade an appropriate phase to investigate our research questions, as pupils have experienced a notable amount of decline in learning motivation by then (e.g., Spinath and Spinath 2005; Weidinger et al. 2017). Consequently, we expected pupils’ NFC to lack ceiling effects and therefore to show enough variance to potentially serve as a predictor. In most German federal states, primary school ends after fourth grade, which marks the transition to secondary education. Teachers’ recommendations and parents’ decisions about which school tracks the pupils are going to attend largely depend on the grades pupils achieve between the end of third grade and fourth grade (Caro et al. 2009). In total, *N* = 565 third-graders (298 girls, 261 boys, 6 with no gender specified; *M*_age_ = 8.40, *SD* = 0.59) in 52 classes from 22 schools in the Ruhr-Area in Germany took part. In the second measurement time (t_2_) in August and September 2022, *n* = 445 children (78.76% of the initial sample) participated. The attrition rate was somewhat higher for children with lower intelligence (*d* = 0.33; see Bergold and Steinmayr 2024), but showed no association with NFC scores.

Teachers and parents were also given a questionnaire, in which they provided information on grades and parental education, respectively. At t_1_, 39 teachers (*M*_age_ = 41.63 years, *SD* = 10.79) took part in the survey. Trained research assistants conducted the pupils’ assessments which took 90 min at both measurement points. Children who only participated in t_1_ remained in the sample to retain maximum information.

### 2.2. Materials

#### 2.2.1. Need for Cognition

We assessed the pupil’s NFC using the NFC scale for primary school children developed by Preckel and Strobel (2017), which is based on the original scale by Cacioppo et al. (1996). Its validity was shown by Keller et al. (2019). The scale comprises 14 items (e.g., “I like doing tasks where I have to think a lot”) with a four-point Likert scale (1 = does not apply at all, 4 = fully applies; displayed as age-appropriate, different-sized stars). The internal consistency in our sample at t_1_ was α = 0.85 and ω = 0.86.

#### 2.2.2. Fluid Intelligence

Fluid intelligence was measured using the German short version of the Culture Fair Intelligence Test (CFT 20-R; Weiß 2006). It contains four subtests (series completion, classifications, matrices, and topological reasoning) with a total of 56 items (α = 0.77 and ω = 0.60^1^).

#### 2.2.3. School Grades

Teachers provided the pupils’ grades in German and in math at both measurement occasions. More precisely, these were the report marks the pupils would have received at the respective time of measurement. This information was available for 50 to 60% of the initial sample (see Table 1).

In Germany, grades range from 1 to 6, with 1 indicating outstanding and 6 indicating insufficient performance. Grades were recoded so that better performance was indicated by higher grades.

#### 2.2.4. Parental Education

Parents reported their highest educational level (1 = no graduation, 2 = lower secondary education, 3 = intermediate secondary school certificate, 4 = entrance qualification for university of applied sciences, 5 = entrance qualification for university). We used the highest value of either parent when both of them reported their educational level.

### 2.3. Statistical Analyses

In a first set of analyses, we performed hierarchical regressions to test whether Gf (H1a), NFC (H2a), and their interaction (RQ1) would predict grades at t_1_, that is, cross-sectionally. In Step 1, parental education and Gf were entered into the model. In Step 2, NFC was added. In Step 3, the interaction between the two centered manifest predictors was entered.

To investigate changes in German and math grades between t_1_ (third grade) and t_2_ (fourth grade), we first applied univariate latent change score models (LCSM) without predictors. We then included latent Gf and latent NFC as predictors of the grades’ changes to test H1b and H2b. Latent NFC was modelled using a single indicator, which had been corrected for unreliability (using internal consistency; see also Bergold and Steinmayr 2024). The indicators of latent Gf were the four subtests of the CFT 20-R. In the last step, we also integrated the latent interaction between Gf and NFC into the models (Klein and Moosbrugger 2000) to examine whether it was a significant predictor of change in school grades (RQ1). We followed the unconstrained approach by Marsh et al. (2004) to model the latent interaction term. That is, we multiplied each indicator of Gf (i.e., the four subtests) with the single indicator of NFC to define the indicators of the latent interaction (error covariances were not allowed). This resulted in four indicators of the latent interaction. Finally, we included parental education as a control variable in the LCSM.

Both German grades and math grades exhibited notable variance at the class level (11.7% and 2.9%, respectively). To account for the pupils being nested in classes, we specified a multilevel structure for all analyses. We centered all predictors at the group mean to obtain unbiased estimations of the within-class effects. All types of missing data (see Section 2.1) were handled using the full information maximum likelihood approach. All analyses were performed using Mplus 8.5.

## 3. Results

Descriptive statistics and correlations between the study variables can be found in Table 2. All correlations were statistically significant and within the expected range based on both theory and previous findings. The control variable parental education correlated significantly with pupils’ Gf (Spearman’s ρ = 0.31, *p* < .001) and with grades at both measurement occasions (ρ between 0.38 and 0.44), but not with NFC.

The results of the hierarchical regression (Table 3) show that even when controlling for parental education, Gf was significantly related to both German and math grades at t_1_ (H1a). Furthermore, NFC was related to grades in both school subjects beyond the effect of Gf (H2a). With regard to RQ1, the interaction between Gf and NFC did not explain additional variance in the school grades.

As a robustness check, we also entered Gf and NFC in a different order into the hierarchical regression. When Gf was entered into the regression after NFC, it still remained incrementally predictive for both German and math grades. The results of the univariate LCSM for math and German grades (see Table 4) show that there was a mean change in both grades (i.e., the pupils had slightly worse grades at the second measurement time than at the first). However, there was significant interindividual variability in this change.

The next steps were to integrate the predictors Gf and NFC and their interaction into the LCSM. The results are shown in Table 5. In both the models with and without the latent interaction, Gf was significantly associated with change in math grades (β = 0.205 and β = 0.200, respectively, *p* < .05), but not with change in German grades. This means that higher intelligence buffered the decline in math grade. NFC was not related to the change in either subject. The same was true for the interaction of Gf and NFC.

When integrating the control variable parental education into the LCSM, the most important difference was that the relation between Gf and changes in math grades was no longer significant (see Table 6).

## 4. Discussion

Intelligence is undoubtedly one of the strongest predictors of academic achievement, including school grades (e.g., Roth et al. 2015). NFC has been shown to play an incremental role for academic achievement in samples at higher educational levels (e.g., Preckel 2014). However, its impact on primary school grades has only scarcely been investigated thus far. One aim of the present study was therefore to analyse the unique contributions of Gf and NFC to school grades and their changes from third to fourth grade. Furthermore, we explored whether Gf and NFC would interact in predicting school grades and their changes.

### 4.1. Effects of Intelligence

The cross-sectional results showed that Gf was significantly associated with both German and math grades. The meta-analysis by Roth et al. (2015) on cross-sectional studies has also demonstrated that intelligence is highly relevant to school grades as early as primary school age, even though correlations at that stage were lower than at higher grade levels. In addition, intelligence has been shown to be the strongest predictor of GPA as early as in primary school (Laidra et al. 2007). Steinmayr and Kessels (2024) have recently also highlighted the relevance of intelligence for predicting academic achievement in primary school. Our cross-sectional finding on Gf, therefore, aligns with previous cross-sectional research.

According to the longitudinal analyses, Gf predicted change in math grades, but not in German grades. The general decline in math grades appeared to be smaller for pupils with higher intelligence than for those with lower intelligence. It seems that their cognitive capacities helped them to mitigate the decline in math grades (or that some pupils experienced consistency or even an increase in math grades, while most other pupils experienced decline). In summary, H1 can be confirmed for math grades, but not fully for German grades. Although we did not expect that Gf does not predict change in German grades, this finding might at least partly be explained by the fact that general intelligence (as well as its most important ingredient Gf) is more closely tied to mathematical abilities and math grades than to language abilities and language grades (Roth et al. 2015). Therefore, fluid ability might have a stronger influence on the development of math achievement than on language achievement. When the control variable parental education was considered in the LCSM, the significant relation between intelligence and change in math grades vanished. This effect could be interpreted in the sense that the more intelligent children had more educated parents (which might also be due to a higher intelligence) that helped them to succeed in school as parental involvement in their children’s education is generally stronger in higher educated families (Benner et al. 2016; Roksa and Potter 2011), Furthermore, meta-analyses indicate that parental involvement has a greater impact on grades than on standardized test scores in primary school (Jeynes 2005). The stronger influence on grades may be due to their reflection of various aspects of student performance, such as homework completion and engagement (Steinmayr et al. 2014) and NFC (Strobel et al. 2024). Furthermore, more educated parents tend to have higher academic aspirations for their children and prefer that they attend an academic-track school (Gymnasium) after fourth grade (Astleithner et al. 2023). Teachers’ recommendations for the school track following primary school largely depend on the grades pupils achieve between the end of third grade and fourth grade (Caro et al. 2009). In the federal state where the pupils were tested, parents ultimately make the final decision regarding their children’s school track. However, their choices are significantly influenced by teachers’ recommendations (Jonkmann et al. 2010). Therefore, it is possible that more educated parents place greater emphasis on maintaining grade levels, which may have contributed to the lower decline observed.

### 4.2. Effects of Need for Cognition

With respect to H2, NFC was associated with school grades within one measurement occasion above and beyond intelligence. The cross-sectional relation between NFC and school grades was higher than what Luong et al. (2017) have presented in their study on Finnish pupils at a comparable age (β = −0.030, *p* = .502). Keller et al. (2019) have also demonstrated the incremental value of NFC in explaining variance in school grades in primary school, even though this increment was beyond motivational variables, whereas intelligence was not tested. Our findings are furthermore in line with results from two studies concentrating on early secondary school age, in which NFC was associated with school grades above intelligence (Lavrijsen et al. 2023; Preckel 2014).

However, when it came to the prediction of change in grades, NFC had no predictive value in either subject. H2 could therefore only be confirmed for the cross-sectional analyses, not for the longitudinal analyses. This result is not in line with the findings by Strobel et al. (2024), who showed that NFC predicted change in GPA in secondary pupils, but it needs to be noted that the sample (eleventh grade in the academic track in Germany) differs from our primary school sample. It might be that the role of NFC for explaining change variance becomes more prominent at the end of adolescence, and maybe even more so in higher-educated samples. However, the effect of intelligence was not tested in the study by Strobel et al. (2024).

Since research on the connection between NFC and school grades at primary school age is still relatively scarce, our results need to be replicated in future studies. According to our findings, NFC does not seem to be as relevant in primary school as it is at later stages of the educational career, at least with regard to changes in educational achievement. Bergold and Steinmayr (2024) have argued that young children might not have that much freedom to choose their cognitive challenges according to their investment traits as they have later in life. Therefore, NFC might not be as important for the development of academic achievement as in later stages. Instead, other characteristics might play a bigger role in shaping academic success at a young age, such as conscientiousness and agreeableness (Laidra et al. 2007).

### 4.3. Interaction Effect

When exploring RQ1, we found no interaction between Gf and NFC for the prediction of neither German nor math grades, nor their changes. This finding contradicts the proposition of investment theories that personality traits and motivational variables moderate the degree to which investment in Gf actually results in higher Gc, including academic achievement (Ackerman 1996; Cattell 1987; Ziegler et al. 2012). However, taken together, the empirical evidence remains inconsistent. On one hand, there are studies like ours that do not find interaction effects between intelligence and NFC or related constructs when predicting academic achievement or other indicators of Gc (Bergold and Steinmayr 2018; Lavrijsen et al. 2023; Scherrer et al. 2024). On the other hand, there are studies that have revealed interaction effects. For example, Strobel et al. (2019) found a compensatory interaction between intelligence and NFC in the retrospective prediction of GPA among university students (see also Zhang and Ziegler 2015; Ziegler et al. 2012). Direct comparisons between these studies appear difficult, as they differ in many respects, such as age group, selectivity of the sample, cultural context, control variables, time intervals, etc. Given that research on interaction effects is still scarce, we advocate for conducting further studies using different methodological approaches to one day be able to meta-analyse the findings and identify conditions under which interaction effects do or do not occur.

### 4.4. Limitations and Future Directions

Our study has some limitations that need to be considered when interpreting its findings. Although grades are among the most important indicators of school success in Germany, it is a limitation that we did not focus on objectively measured school performance. Besides actual performance, grades are influenced by several distant (e.g., socio-economic background, gender) and proximal variables (e.g., students’ ability self-concept, personality) (e.g., Steinmayr and Kessels 2024; Spinath et al. 2010). These variables also impact how teachers evaluate a student’s performance (see Steinmayr et al. 2014). Gf might have remained a significant predictor if we had focused on change in pupils’ performances on standardized achievement tests. Comparably, results might have differed if we had analysed Gc instead of Gf or as an addition to Gf. Both issues could be addressed by future studies.

A further weakness related to the grades might be the relatively high missing rates for the school grades provided by teachers. However, the missing grades were not specific for individual children but stemmed from teachers who generally did not participate in the survey.

It is also noteworthy that our data were collected right after the COVID-19 pandemic that came along with school lockdowns, also in Germany (for further details on the exact schedule of the survey with regard to the lockdowns, see Bergold and Steinmayr 2024). We cannot rule out the possibility that these lockdowns and other restrictions could be one explanation for why grades became slightly worse in the fourth grade as compared to the third grade. Recent research has demonstrated that the school lockdowns hindered learning (Di Pietro 2023) and possibly pupils’ intellectual development (Breit et al. 2023). However, grades in third grade were already obtained after the school lockdowns. Another explanation might be that teachers became stricter in grading in fourth grade, as fourth grade is the last school year before the transition to one of the secondary school tracks. Whereas grading in third grade might additionally be guided by pedagogical intentions (e.g., supporting the pupils, motivating them, etc.), grading in the last school years might be more strongly guided by a focus on performance in the face of the upcoming decision of who will be able to succeed in the academic track and who will not. This thought is supported by the findings in another primary school sample in which a decline in math grades was found in pre-pandemic times (Weidinger et al. 2017). In any case, this limitation does not threaten our conclusions, because we were interested in predicting interindividual differences in change.

Finally, the design of the study was correlational, which limits our ability to draw conclusions about causal relations between the variables of interest. Additionally, only two measurement points were available, which does not allow for separating the relations within individuals from the relations between individuals (Hamaker et al. 2015). It might thus be worthwhile for upcoming research to include more than two measurement occasions, which would also enable modelling the development of grades over a longer time.

### 4.5. Conclusions

The present study was the first to investigate the roles of Gf and NFC with regard to changes in school grades in primary school. An additional focus was laid on a potential interaction effect between Gf and NFC, addressing the question of whether pupils benefit from being intelligent *and* enjoying cognitive stimulation or whether high NFC can make up for lower intelligence. We found no indication of any interaction between Gf and NFC in predicting school grades and their changes. In contrast to findings from older samples, NFC does not seem to play a comparably relevant role in primary school, at least with regard to changes in school grades. Reasons for that might lie in the status of intellectual and motivational development at that age or other determinants of educational success in primary school. Our findings speak against the role of investment traits as proposed by investment theories, at least for primary school children. Investing energy in fostering investment traits such as NFC should therefore be carefully considered. Nevertheless, as NFC has been shown to be beneficial at later stages of education, fostering NFC may still have lagged effects.

## Figures and Tables

**Table 1:** Sample sizes for school grades provided by teachers.

Measurement Time	Math	German
t_1_	293 (51.8%)	338 (59.9%)
t_2_	283 (50.0%)	336 (59.4%)

**Table 2:** Descriptive statistics and correlations of observed scores.

	M	SD	Correlations						
			1	2	3	4	5	6	7
1 NFC t_1_	3.09	0.56		0.56 ***	0.16 ***	0.26 ***	0.22 ***	0.20 ***	0.16 **
2 NFC t_2_	3.03	0.55			0.16 ***	0.25 ***	0.27 ***	0.18 ***	0.28 ***
3 Gf t_1_	23.64	6.43				0.41 ***	0.43 ***	0.48 ***	0.42 ***
4 Math grade t_1_	2.51	1.09					0.66 ***	0.69 ***	0.48 ***
5 Math grade t_2_	2.64	0.97						0.59 ***	0.71 ***
6 German grade t_1_	2.72	1.07							0.77 ***
7 German grade t_2_	2.74	0.99							

*Note.* Grades were recoded so that higher values indicate higher achievement. NFC = need for cognition. Gender: 0 = male, 1 = female. *** *p* < .001, ** *p* < .01.

**Table 3:** Results of the hierarchical regression for German and math grades at t_1._

Outcome	Predictor	*B*	β	*p*	*R* ^2^	Δ*R*^2^
Math grade at t_1_	Education	0.192	0.169	.112	0.162	
	Gf	0.059	0.319	<.001		
	Education	0.190	0.167	.081	0.239	0.077
	Gf	0.051	0.279	<.001		
	NFC	0.607	0.281	<.001		
	Education	0.189	0.166	.083	0.239	0.000
	Gf	0.051	0.279	<.001		
	NFC	0.619	0.286	.002		
	Gf × NFC	−0.005	−0.015	.859		
German grade at t_1_	Education	0.151	0.126	.178	0.151	
	Gf	0.062	0.331	<.001		
	Education	0.145	0.120	.123	0.199	0.048
	Gf	0.056	0.299	<.001		
	NFC	0.495	0.222	.003		
	Education	0.145	0.121	.126	0.200	0.001
	Gf	0.056	0.299	<.001		
	NFC	0.489	0.219	.007		
	Gf x NFC	0.003	0.008	.900		

*Note.* Education = The highest school-leaving certificate of the parents.

**Table 4:** Latent change in math and German grades.

	Δ (SE)	σΔ (SE)
Math	−0.178 (0.075) *	0.500 (0.067) ***
German	−0.137 (0.074)	0. 369 (0.059) ***

*Note.* Unstandardized solution. Grades were recoded so that higher grades display higher achievement. *** *p* < .001, * *p* < .05.

**Table 5:** Regression weights from the predicted latent change score models for math and German Grades.

Predictors	Δ Math Grades	Δ German Grades
	Model without latent interaction	
Gf t_1_	0.205 (0.101) *	0.065 (0.104)
NFC t_1_	0.014 (0.057)	−0.048 (0.061)
	Model fit	
	Χ^2^(df) = 9.990 (11); CFI > 0.999; RMSEA < 0.001; SRMR = 0.021	Χ^2^(df) = 12.457 (11); CFI = 0.997; RMSEA = 0.015; SRMR = 0.023
	Full model with latent interaction	
Gf t_1_	0.200 (0.099) *	0.067 (0.089)
NFC t_1_	−0.009 (0.067)	−0.062 (0.068)
Gf × NFC	0.070 (0.096)	0.067 (0.095)
	Model fit	
	Χ^2^(df) = 28.157 (37); CFI > 0.999; RMSEA < 0.001; SRMR = 0.030	Χ^2^(df) = 36.320 (37); CFI > 0.999; RMSEA < 0.001; SRMR = 0.033

*Note.* Standard errors in parentheses. * *p* < .05. CFI = comparative fit index; RMSEA = root mean square error of approximation; SRMR = standardized root mean square residual.

**Table 6:** Regression weights from the predicted latent change score models for math and German Grades, including parental education.

Predictors	Δ Math Grades	Δ German Grades
	Model without latent interaction	
Gf t_1_	0.150 (107)	0.019 (0.091)
NFC t_1_	0.027 (0.056)	−0.037 (0.061)
Education	0.234 (0.084) **	0.195 (0.075) **
	Model fit	
	Χ^2^(df) = 13.959 (14); CFI > 0.999; RMSEA < 0.001; SRMR = 0.023	Χ^2^(df) = 14.975 (14); CFI = 0.998 RMSEA = 0.011; SRMR = 0.024
	Full model with latent interaction	
Gf t_1_	0.145 (0.105)	0.018 (0.105)
NFC t_1_	0.014 (0.066)	−0.045 (0.070)
Gf × NFC	0.039 (0.090)	0.042 (0.092)
Education	0.232 (0.085) **	0.197 (0.076) *
	Model fit	
	Χ^2^(df) = 32.837 (43); CFI > 0.999; RMSEA < 0.001; SRMR = 0.030	Χ^2^(df) = 39.668 (43); CFI > 0.999; RMSEA < 0.001; SRMR = 0.032

*Note.* Standard errors in parentheses. Education = The highest school-leaving certificate of the parents. ** *p* < .01, * *p* < .05. CFI = comparative fit index; RMSEA = root mean square error of approximation; SRMR = standardized root mean square residual.

## Data Availability

The raw data supporting the conclusions of this article will be made available by the authors on request.

## Note

1 Cronbach’s α was based on all 56 test items. McDonald’s ω was based on the 4 subtests as indicators. McDonald’s ω uses a factor analytical approach. Due to some of the item correlations being zero, the 56 items of the CFT-R 20 cannot be used as indicators.
